# Galactosylated Liposomes for Targeted Co-Delivery of Doxorubicin/Vimentin siRNA to Hepatocellular Carcinoma

**DOI:** 10.3390/nano6080141

**Published:** 2016-07-30

**Authors:** Hea Ry Oh, Hyun-Young Jo, James S. Park, Dong-Eun Kim, Je-Yoel Cho, Pyung-Hwan Kim, Keun-Sik Kim

**Affiliations:** 1 Department of Biomedical Laboratory Science, Konyang University, Daejeon 35365, Korea; hearyoh10@gmail.com (H.R.O.); hyun02for@hanmail.net (H.-Y.J.); 2 Department of Medicine, New York University School of Medicine, New York, NY 10016, USA; james.park@nyumc.org; 3 Department of Bioscience and Biotechnology, Konkuk University, Seoul 05029, Korea; kimde@konkuk.ac.kr; 4 Department of Biochemistry, College of Veterinary Medicine, Seoul National University, Seoul 08826, Korea; jeycho@snu.ac.kr

**Keywords:** targeted liposomes, doxorubicin, vimentin siRNA, combination therapy, co-delivery, hepatocellular carcinoma

## Abstract

The combination of therapeutic nucleic acids and chemotherapeutic drugs has shown great promise for cancer therapy. In this study, asialoglycoprotein receptors (ASGPR) targeting-ligand-based liposomes were tested to determine whether they can co-deliver vimentin siRNA and doxorubicin to hepatocellular carcinoma (HCC) selectively. To achieve this goal, we developed an ASGPR receptor targeted co-delivery system called gal-doxorubicin/vimentin siRNA liposome (Gal-DOX/siRNA-L). The Gal-DOX/siRNA-L was created via electrostatic interaction of galactose linked-cationic liposomal doxorubicin (Gal-DOX-L) on vimentin siRNA. Previous studies have shown that Gal-DOX/siRNA-L inhibited tumor growth by combined effect of DOX and vimentin siRNA than single delivery of either DOX or vimentin siRNA. These Gal-DOX/siRNA-Ls showed stronger affinity to human hepatocellular carcinoma cells (Huh7) than other cells (lung epithelial carcinoma, A549). These liposomes also have demonstrated that novel hepatic drug/gene delivery systems composed of cationic lipid (DMKE: *O,O’*-dimyristyl-*N*-lysyl glutamate), cholesterol, galactosylated ceramide, POPC (1-palmitoyl-2-oleoyl-*sn*-glycero-3-phosphocholine), and PEG_2000_-DSPE (distearoyl phosphatidyl ethanolamine) at 2:1:1:1:0.2 (moral ratios) can be used as an effective drug/gene carrier specifically targeting the liver in vivo. These results suggest that Gal-DOX-siRNA-L could effectively target tumor cells, enhance transfection efficacy and subsequently achieve the co-delivery of DOX and siRNA, demonstrating great potential for synergistic anti-tumor therapy.

## 1. Introduction

Due to the complexity of tumorigenesis and difficulty of cancer therapy, combinations of chemotherapeutic drugs with other treatment modalities, like nucleic acid, have shown great promise in cancer therapy [1]. In the past few decades, there has been a growing scientific interest to develop functional drug/gene delivery systems derived from natural resources with good biocompatibility, controllable, and highly efficient delivery capability [2,3]. However, it has been difficult to find a stable drug and gene delivery system in blood circulation. The delivery of the drug and gene often show non-specific distribution in the subjects and the iatrogenic immune response to the surrounding normal tissue seriously limited the clinical application of drug/gene combination therapy [4]. Therefore, it is necessary to design an ideal multifunctional nano-delivery system for co-delivery of the drug and gene together, overcoming the above mentioned problems.

Doxorubicin (DOX) is a potential anticancer drug that commonly used in chemotherapy for cancer treatment. However, the clinical uses of DOX are restricted largely due to limited tissue specificity and serious cardiotoxic effects resulting from the generation of free radicals and lipid peroxidation. In order to reduce the DOX toxicity, a liposomal formulation was used. However, the efficacy of DOX treatment was not enhanced greatly by liposomal delivery. Doxorubicin (DOX) is used widely to treat diverse types of cancer, yet its effectiveness is hampered by the existence of drug-resistant cancer cells [5]; thus, it is necessary to combine gene therapy with DOX to achieve a synergistic combination therapeutic effect.

Small interfering RNAs (siRNAs) have become the main focus of the recent therapeutic applications. Hence, siRNA therapeutics have demonstrated potential as a more personalized approach in the treatment of many life threatening disease caused by faulty protein expression, such as malignancies and fibrosis [6]. Vimentin, a major constituent of the intermediate filament (IF) family of proteins, is known to maintain cellular integrity and provide resistance against stress. The increasing of vimentin expression has been reported in diverse epithelial cancers, including breast cancer, prostate cancer, lung cancer, and other types of cancers. In our previous study, we have shown that downregulation of vimentin by Sendai F/HN viroplexes caused vimentin siRNA to inhibit the growth of hepatocellular carcinoma in vitro, which suggests again that vimentin is a potential target for gene therapy [7]. However, for developing an effective delivery strategy, the feasibility of vimentin targeted therapy needs to be investigated.

Combination therapy with chemotherapeutics and siRNAs has been studied as an alternative way of achieving potentiated anticancer activity. Several investigators have created delivery systems based on liposomes or nanoparticles for co-delivery of anticancer chemotherapeutics and siRNAs. Some of the reported delivery system include: mesoporous silica nanoparticles for deliver Bcl-2 siRNA and DOX to effective treatment of cancer cells [8] and cationic liposomes for co-delivery of doxorubicin (DOX) and siRNA targeting multi-drug resistance (MDR) protein to boost anticancer effect in lung cancer cells [9]. Among the various types of delivery systems, the liposomal delivery system has attracted considerable attention. Compared with free chemical drugs, liposomes can help to prolong bioavailability time in circulation, and this help to target MDR-enhanced tumor localization [10,11]. Liposomes can also reduce the drug related adverse effects, such as the cardiotoxicity of DOX [12,13]. Over two decades, studies have been focused on the development of novel liposomal therapeutic agents that have high encapsulation efficiency and enhanced passive targeting efficiency.

In recent years, many studies have been focused on different targeting delivery systems, such as targeting systems for transferrin receptors [14], folate receptor targeting delivery system [15], RGD conjugating drug delivery system [16], and asialoglycoprotein receptor-mediated hepatocyte targeting [17]. The asialoglycoprotein receptor (ASGPR) is primarily expressed on hepatocytes and minimally on extrahepatic cells. At this point, to induce liver cancer-specific gene delivery, galactose, or *N*-acetylgalactosamine can use as a targeting ligand. Using this ligand for ASGPR-mediated targeting can be manufactured with a variety of therapeutic carriers as target moiety [18,19]. This makes it specifically attractive for receptor-mediated drug or gene delivery with minimum concerns of toxicity. ASGPR facilitates internalization by clathrin-mediated endocytosis and exhibits high affinity for carbohydrates, specifically galactose. Popular ligands for ASGPR mediated targeting are galactose-bearing glycoproteins, glycopeptides, and galactose modified polymers and lipids [20,21].

**Scheme 1:**
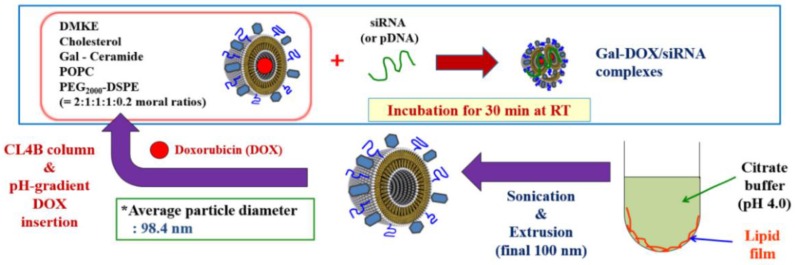
Schematic illustration of formation of Gal-DOX/siRNA-L (galactosylated-doxorubicin/ vimentin siRNA liposome). Firstly, doxorubicin (DOX) was encapsulated in cationic galactosylated liposomes by the pH-gradient insertion method. Subsequently, galactosylated liposomal DOX (Gal-DOX-L) and siRNA were co-loaded (Gal-DOX/siRNA complexes) by electrostatic attraction.

In the present study, asialoglycoprotein receptors (ASGPR) targeting-ligand-based liposomes were prepared by cationic lipid (DMKE), POPC, cholesterol, PEG_2000_-DSPE, and galactosylated ceramide to co-deliver DOX and vimentin siRNA, expecting to target hepatic carcinoma sites, enhance transfection efficiency, and realize the optimal combination therapy (as shown in Scheme 1). These liposomes have shown higher cancer cell cytotoxicity effects triggered by gal- doxorubicin/vimentin siRNA liposome (Gal-DOX/siRNA-L) than each single treatment. In addition, the cellular targeting and in vivo localization of Gal-liposomes ware also observed by fluorescence microscopy and luciferase assay, respectively. Therefore, we believe that the Gal-liposomal particles are a useful hepatic targeting system for co-delivery of drug and gene.

## 2. Results

### 2.1. Characterization of Liposomes

The physicochemical properties of Gal-DOX-L (cationic liposomes) or Gal-DOX/siRNA-L (lipoplexes) are presented in Table 1. Galactose linked-cationic liposomal doxorubicin (Gal-DOX-L) had a mean diameter of 98.4 ± 2.2 nm and zeta potential of 35.7 mV, whereas Gal-DOX/siRNA-L complexes had a relatively larger diameter of 135.21 ± 22 nm and a lower zeta potential of 9.4 mV. The optimal formulations of Gal-DOX/siRNA-L with nitrogen to phosphate (N/P) ratios (N = arginine amino groups of lipid; P = siRNA phosphate groups) of 1, 3, 6, and 9 and they were evaluated to find the most optimal N/P ratio for preparation. The entrapment efficiency of siRNA in Gal-DOX/siRNA-L formulation by remote loading was greater than 85% at N/P ratio of 9 with an encapsulation capacity (Table 1).

**Figure 1:**
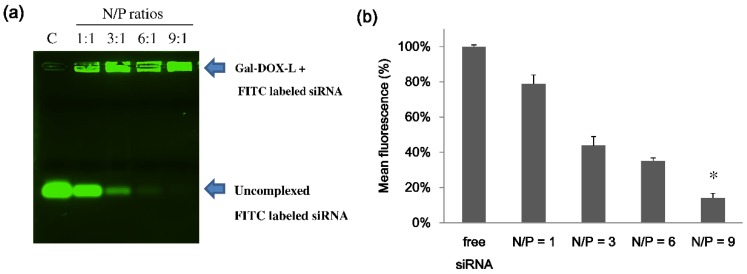
Gel retardation analysis and fluorescence intensities measurement of Gal-DOX/siRNA-L complexes at various N/P ratios. (**a**) Gel retardation assay of different formulations of siRNA, containing FITC-siRNA (200 nM) per sample, on a 2% TAE (Tris Acetate EDTA) agarose gel at 100 V for 30 min, then investigated by UV (Ultraviolet) illuminator; and (**b**) fluorescence intensities measured uncomplexed free FITC-labeled siRNA at varying N/P ratios in the gel by a FUSION SL chemiluminescence analyzer and software (VILBER, Suarlée, Belgium). (* *p* < 0.05 vs. N/P = 6; mean ± SD; *n* = 3 replicates/group).

Gal-DOX/siRNA-L formations with N/P ratios of 1, 3, 6, and 9 were analyzed for their ability to retain siRNA cargo. Freshly-prepared test samples were tested via gel retardation assay to visually determine the presence of uncondensed FITC (fluorescein isothiocyanate)-labeled siRNA (Figure 1a). In lanes 4 and 5 (N/P = 6 and 9), the free FITC-siRNA band has been significantly reduced. A separate aliquot of the same test solution was further analyzed by chemiluminescence analyzer to quantify uncomplexed FITC-labeled siRNA in the samples; data are expressed as percentage of free FITC-labeled siRNA control (Figure 1b). FITC-labeled siRNA band with an intensity close to that of the free siRNA control was detected with Gal-DOX/siRNA-L formation of N/P ratio = 9 (~86% of untreated free siRNA control) and quantified the encapsulation efficiency (EE) of siRNA in Gal-DOX/siRNA-L complexes by chemiluminescence analyzer.

### 2.2. Specific Binding of Gal-DOX-L to Hepatocellular Carcinoma (HCC) Cells

Ligand-mediated targeting delivery system is one of the most exciting areas for improvement of liver-specific drug delivery. In order to achieve highly effective liver-targeting delivery systems, galactose- (or lactose-) mediated liposomes previously designed and evaluated luciferase expression level using pDNA (luciferase expression vector) for targeting delivery efficiency in vivo (Figure S1). The ligand mediated liposomal delivery systems were also evaluated to optimize of the transfection efficiency in vitro (Figures S2 and S3). In this study, the galactose-mediated liposome was designed based on these results that the transfection efficiency of galactose-mediated liposomes was higher compared with that of lactose-mediated liposomes.

**Figure 2:**
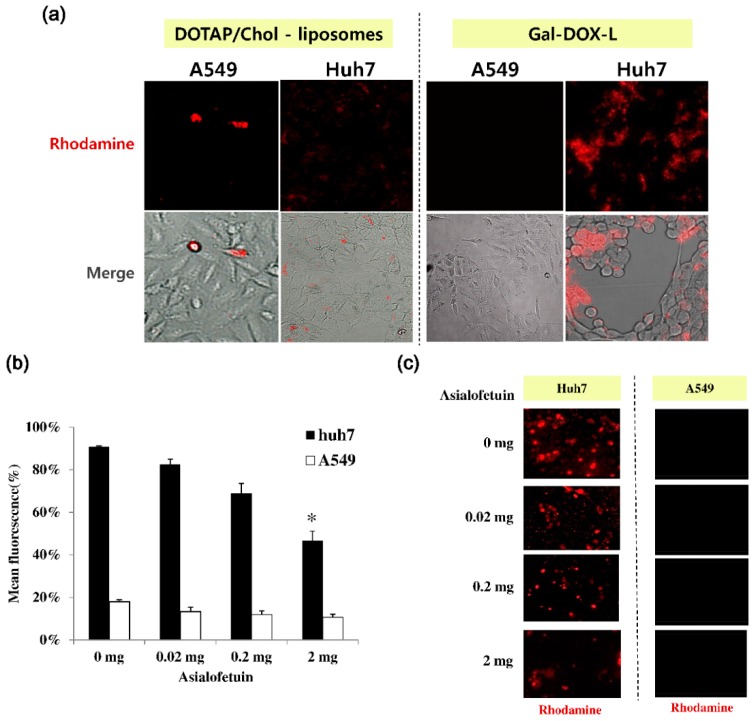
Specific binding of the Gal-DOX-Ls to human hepatocarcinoma Huh7 cells. (**a**) A549 and Huh7 cells were incubated with either plane liposomes (DOTAP/Chol liposomes) or Gal-DOX-Ls for 15 min in culture media. These liposomes having the rhodamine-conjugated DOPE lipids (red color) were observed on the cell surface by a fluorescence microscope (JuLi-Smart Fluorescence Cell Imager, NanoEnTek Inc., Seoul, South Korea). (**b**) For asialofetuin (AF) inhibition, AF in culture media added as increasing concentration for 30 min before the treated of Gal liposome (20 μg). Cells were analyzed after 30 min last treatment of Gal liposome. Red fluorescence color in each cells were investigated by a Tali image-based cytometer (Thermo Fisher Scientific Inc., Waltham, MA, USA). (**c**) These liposomes having the rhodamine-conjugated DOPE lipids were observed on the cell surface by an Axio Zeiss A1 Imager compound microscope (Carl Zeiss, Oberkochen, Germany). Error bars represent standard deviation of three independent experiments. * *p* < 0.01 when compared to with no treatment (asialofetuin, 0 mg).

The Gal-DOX-Ls containing fluorescent dye-conjugated phospholipid (Rho-DOPE) were used to evaluate specific biding to the surface of tumor cells overexpressing asialoglycoprotein receptors (ASGPR). Gal-DOX-Ls were added to the ASGPR-positive human hepatocarcinoma Huh7 cells and ASGPR-negative human pulmonary carcinoma A549 cells in the condition of whole media with serum. Specific binding of Gal-DOX-Ls were observed only in Huh7 (ASGPR-positive), but not in A549 cells (ASGPR-negative) by the fluorescence from the rhodamine-conjugated liposomes (Figure 2a). To reevaluate the evidence of ASGPR (+) or ASGPR (‒) of both cells (Huh7 and A549), these cells treated with asialofetuin (a natural ligand for asialoglycoprotein receptors) before the treatment of Gal-liposomes avoiding DOX (Figure 2b,c). The Gal-liposomes containing rhodamine fluorescent dye-conjugated phospholipid (Rho-DOPE) were used to evaluate specific biding to the surface of tumor cells overexpressing ASGPR (Figure S4). Specific binding of the Gal-liposomes showed the dose-dependent binding inhibition only in Huh7 (ASGPR-positive cells), but not in ASGPR-negative cells, A549 (Figure 2c). These results indicate that the galactose conjugated to the liposome effectively targeted to the ASGPR-expressing hepatocellular carcinoma cells.

### 2.3. Cytotoxicity of Gal-DOX-L

**Figure 3:**
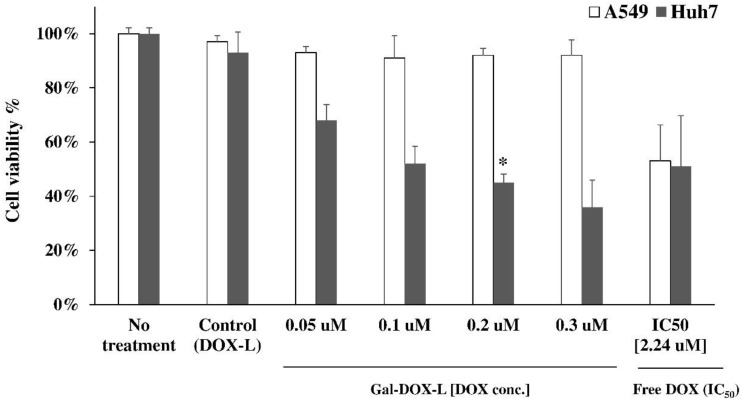
Cytotoxic effect of Gal-DOX-L formation to Huh7 cells. Cell viability was measured after A549 cells and Huh7 cells were incubated with various formations of DOX tor 48 h. The cells were treated with free DOX and DOX-encapsulated in Gal-liposomes (Gal-DOX-L), where each formation was adjusted to contain various amount of DOX. As a control liposome, cells were also treated with liposomal doxorubicin (DOX-L [0.2 μM]) lacking galactose. No treatment means that cells were not treated. Data are represented as the mean ± standard deviation (*n* = 3). * *p* < 0.05 compared with Free DOX (IC_50_) treatment.

The cytotoxicity of Galactose linked-cationic liposomal doxorubicin (Gal-DOX-L) in A549 cells (ASGPR-negative) and Huh7 cells (ASGPR-positive) was studied using MTT (3-(4,5-dimethylthiazol-2-yl)-2,5-diphenyltetrazolium bromide) assay. The cells were treated with different DOX concentration formations. The half-maximal inhibitory concentration (IC_50_) values of free DOX and Gal-DOX-L in Huh7 cells are shown in Figure 3. The cytotoxicity of Gal-DOX-L (IC_50_ = 0.2 μM) was significantly greater than that of free DOX (IC_50_ = 2.24 μM). These results suggest that treatment with Gal-DOX-L resulted in significantly greater the cytotoxic effect compared with free DOX on IC_50_ values.

### 2.4. Transfection Effiiency and Cytotocixity of siRNA by Gal-Lipoplexes in Vitro

**Figure 4:**
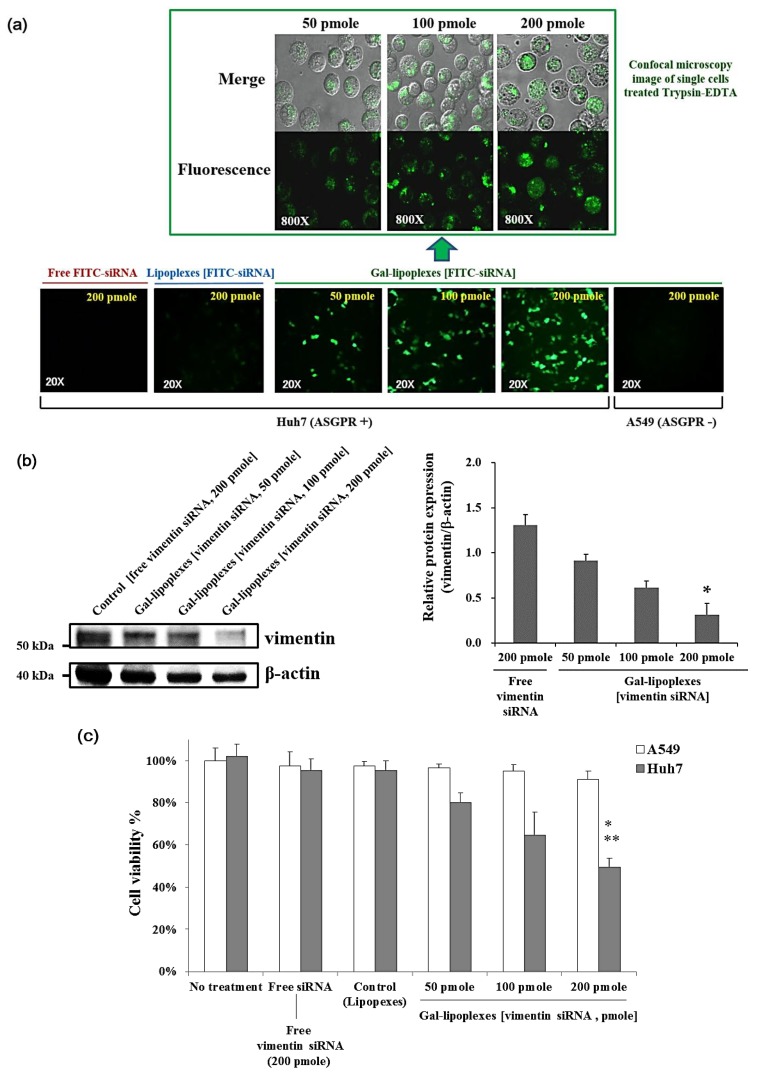
Cell uptake and cytotoxicity by Gal-lipoplexes-mediated siRNA. (**a**) Internalization of FITC-siRNA by Gal-lipoplexes in the ASGPR-expressing Huh7 cells via receptor-ligand mediated endocytosis. A549 cells and Huh7 cells were incubated with Gal-lipoplexes of a various FITC-siRNA concentration (50, 100, and 200 pmole) for 2 h in culture media. FITC-labeled siRNAs were visualized in green at the ASGPR-expressing Huh7 cells; (**b**) inhibition of the expression of vimentin in Huh7 cells by Gal-lipoplexes (vimentin siRNA). Huh7 cells were treated with control free siRNA or Gal-lipoplexes (vimentin siRNA, 50–200 pmole). Total cell lysates (40 μg) of Huh7 cells were subjected to SDS-PAGE (sodium dodecyl sulfate polyacrylamide gel electrophoresis). The separated proteins were analyzed by Western blot method to detect vimentin as described. The β-actin detection was included as a loading control. Values of relative protein expression are expressed as the mean ± standard deviation. * *p* < 0.05 vs. Gal-lipoplexes [vimentin siRNA, 100 pmole]. (**c**) To measure in vitro cytotoxicity of Gal-lipoplexes (vimentin siRNA), Huh 7 cells and A549 cells were incubated with free vimentin siRNA (200 pmole), lipoplexes lacking of galactose (vimentin siRNA, 200 pmole), or Gal-lipoplexes (vimentin siRNA) containing various concentration of vimentin siRNA (50–200 pmole) for 48 h. Cell viability was each monitored by the MTT assay method using EZ-CyTox reagents after 48 h. Data are represented as the mean ± standard deviation (*n* = 4). * *p* < 0.05 vs. Gal-lipoplexes (vimentin siRNA, 100 pmole) in Huh7 cells; ** *p* < 0.01 vs. Gal-lipoplexes (vimentin siRNA, 200 pmole) in A549 cells.

The potential of Gal-liposome devoid of DOX for siRNA transfection was evaluated in vitro on Huh7 (human hepatocarcinoma) cells expressing asialoglycoprotein surface receptors in comparison to free siRNA [22]. The effectiveness of siRNA delivery was observed using a fluorescence microscope and a confocal laser scanning microscope, to determine the cellular uptake efficacy in Huh7 cells which were treated with galactose conjugated cationic lipoplexes with different FITC-labeled siRNA concentration formations of N/P ratio = 9. Generally, Gal-lipoplexes with 200 pmole FITC-siRNA exhibited higher siRNA uptake than the two different concentration of FITC-siRNA at 2 h post-transfection (Figure 4a). This result suggests that the galactose ligand enhances siRNA transfection efficiency at 200 pmole siRNA with galactose-conjugated cationic liposomes.

In order to demonstrate functional siRNA transfection mediated by Gal-lipoplexes, siRNA molecules of vimentin, a member of the intermediate filament structure, were utilized for transfection. Huh7 cells were transfected with vimentin siRNA by Gal-lipoplexes. The cells were transfected under optimized conditions (N/P ratio = 9). After introduction of vimentin siRNA (50, 100, 200 pmole) by Gal-lipoplexes into Huh7 cells for 48 h, suppression of vimentin were confirmed by Western blotting, while the control free vimentin siRNA had no significant effect on endogenous vimentin expression (Figure 4b). The expression of vimentin was reduced by 77% in comparison to the controls (*p* < 0.05). The expression level of vimentin also was reduced by 50% in comparison to Gal-lipoplexes (vimentin siRNA, 100 pmole) (*p* < 0.05). Among the transfected cells, only the cells transfected by Gal-lipoplexes with 200 pmole vimentin siRNA exhibited a reduction the inhibition of vimentin expression (Figure 4b) and the cell viability of IC_50_ at 48 h post-transfection (Figure 4c). Transfection using Gal-lipoplexes reduced the cell viability in 48 h post-transfection by 49%. The treatment of the same amount (200 pmole) of free vimentin siRNA reduced the cell viability in Huh7 cells by 5%. These results show that the transfection of vimentin siRNA by Gal-lipoplexes has anticancer activity using the vimentin siRNA technique.

### 2.5. Combination Therapeutic Effect of DOX and Vimentin siRNA by Gal-DOX/siRNA-L

**Figure 5:**
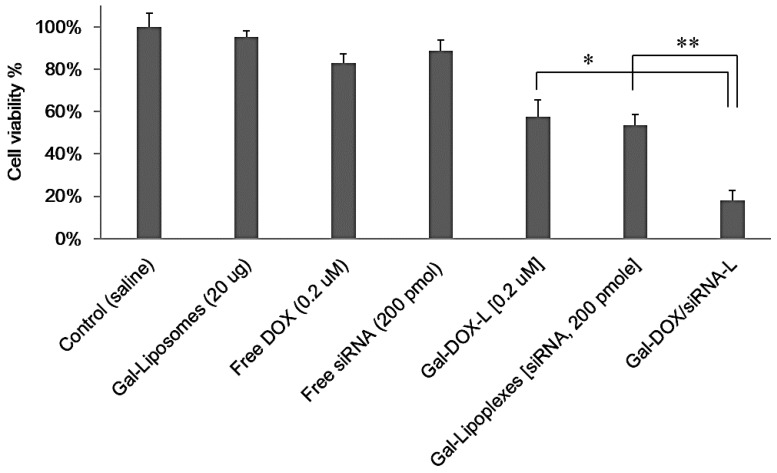
Cytotoxic effects of Gal-DOX/siRNA-L in Huh7 cells. The control group was treated with saline. To measure the cytotoxicity synergic effect of Gal-DOX/siRNA-L, Huh 7 cells were incubated with various formation containing with the final vimentin siRNA concentration of 200 pmole and DOX concentration of 0.2 μM. Cell viability was each monitored by the MTT assay method using EZ-CyTox reagents after 48 h. Data are represented as the mean ± standard deviation (*n* = 3). * *p* < 0.01 vs. Gal-DOX-L (0.2 μM); ** *p* < 0.05 vs. Gal-lipoplexes (siRNA, 200 pmole).

To verify the combination therapeutic effect of Gal-DOX/siRNA-L, the cytotoxic effects of various vimentin siRNA and DOX formations were evaluated in Huh7 cells. Compared with saline treated cells, Gal-DOX/siRNA-L treated cells showed significant cell growth inhibition or cell death (Figure 5). After 48 h post-transfection, almost half of the Huh7 cells were diminished by Gal-DOX-L (0.2 μM) or Gal-lipoplexes (siRNA, 200 pmole) treatment, whereas free DOX (0.2 μM) or free vimentin siRNA (200 pmole) treated Huh7 cells survived well. As expected, Gal-DOX/siRNA-L killed 82% Huh7 cells much higher than Gal-DOX-L (0.2 μM) and Gal-lipoplexes (siRNA, 200 pmole) (Figure 5). Together, these data showed that Gal-DOX/siRNA-L displayed higher cytotoxicity than both Gal-DOX-L and Gal-Lipoplexes (siRNA). These data suggest clearly that DOX and vimentin siRNA delivered by Gal-DOX/siRNA-L showed enhanced cytotoxic effects in vitro.

### 2.6. Biodistribution and Antitumor Activity by Gal-DOX/siRNA-L in Tumor-Bearing Mice

**Figure 6:**
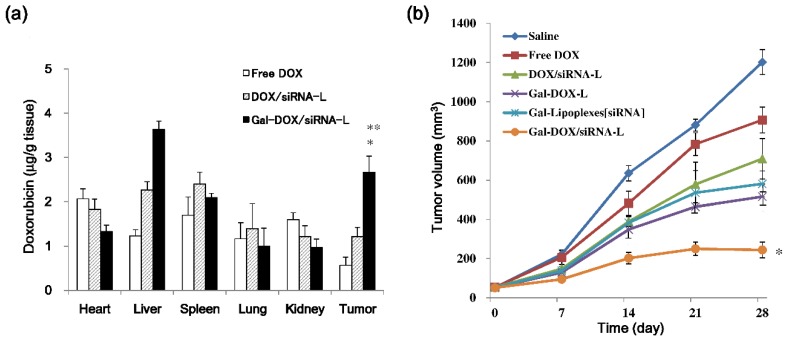
Biodistribution and in vivo anti-tumor efficacy of Gal-DOX/siRNA-L in xenograft nude mice. (**a**) The mice were injected via tail vein at a single dose of free DOX (5 mg/kg), DOX/siRNA-L (5 mg/kg DOX, 150 μg/kg siRNA), and Gal-DOX/siRNA-L (5 mg/kg DOX, 150 μg/kg siRNA) (*n* = 4) when the tumors grew to about 400 mm^3^. The tissues were collected at 4 h after the injections. Date are expressed as the mean ± standard deviation (*n* = 4); and (**b**) growth curves of xenograft tumors treated with saline, free DOX, DOX/siRNA-L, Gal-DOX-L, Gal-Lipoplexes (siRNA), and Gal-DOX/siRNA-L by intravenous injection once a week for four weeks (5 mg/kg DOX or/and 150 μg/kg vimentin siRNA). The curves present the changes of tumor sizes from the day of injection (day 0). Results are expressed as mean and standard deviation (*n* = 4). * *p* < 0.05, significant compared with Gal-DOX-L or Gal-lipoplexes (siRNA).

A distribution of doxorubicin (DOX) in normal or tumor tissues was observed in tumor-bearing Balb/c athymic nude mice after 4 h tail vein injection. Both DOX/siRNA-L and Gal-DOX/siRNA-L injections indicated obvious DOX accumulation in liver tissues with reference to other organs compared with free DOX (Figure 6a). However, the accumulation of DOX delivered by DOX/siRNA-L and Gal-DOX/siRNA-L in hearts and kidneys were lower than that of free DOX. In tumor tissues, Gal-DOX/siRNA-L showed even higher DOX uptake than free DOX and DOX-siRNA-L. Accumulation of Gal-DOX/siRNA-L in tumor tissues was 4.8 times higher than that of free DOX and 2.3 times higher than that of DOX/siRNA-L. These results suggested that Gal-DOX/siRNA-L significantly increased the delivery of DOX to liver and hepatic tumor tissue.

For evaluation of anti-tumor therapeutic efficacy, various formations composed of DOX and/or vimentin siRNA were further analyzed in xenograft Huh7 tumor mouse model. After tumors had developed to ~55 mm^3^, we performed comparative efficacy studies by dividing the animals into four groups. Nude mice bearing Huh7 tumors received an intravenous injection of (i) saline; (ii) Free DOX (5 mg/kg DOX); (iii) DOX/siRNA-L (5 mg/kg DOX, 150 μg/kg siRNA); (iv) Gal-DOX-L (5 mg/kg DOX); (v) Gal-Lipoplexes (150 μg/kg siRNA); or (vi) Gal-DOX/siRNA-L (5 mg/kg DOX, 150 μg/kg siRNA) once a week (*n* = 4 mice/group), and the tumor size was then monitored for four weeks. The results showed that tumor size was significantly reduced in the Gal-DOX/siRNA-L group as compared to the non-targeted saline, free DOX, and DOX/siRNA-L (Figure 6b) groups. In the control-saline and free DOX groups, the treatment did not show significant long-term efficacy of tumor size reduction, and the mean tumor sizes at the end of the study for the groups were 1269 ± 139 mm^3^ and 907 ± 66 mm^3^ , respectively (mean ± SEM; *n* = 4). The treatment for the DOX/siRNA-L group was more efficacy (mean tumor size at end point was 709 ± 103 mm^3^) than that for the control and the Free DOX groups, probably due to passive targeting of tumor tissues through the EPR effect. Notably, Gal-DOX/siRNA-L-treated tumors had the smallest sizes (286 ± 45 mm^3^) among these formation groups. These data suggest that DOX and vimentin siRNA co-delivery by Gal-DOX/siRNA-L can potentiate the tumor inhibition and provide improved cancer therapeutic effect.

## 3. Discussion

Vimentin, one of the intermediate filament family proteins, is universally expressed in normal mesenchymal cells to maintain cellular unity. Increasing of vimentin expression in various epithelial cancers has been reported that vimentin in cancer correlates with increased tumor growth, invasion, and poor prognosis [23,24]. For that reason of its over-expression in cancers and its role in mediating tumorigenic events, vimentin provide as an available targeted cancer therapy [25]. Despite these well-characterized correlations, vimentin has not been sufficiently investigated as a new therapeutic target for siRNA. In particular, there are no published studies showing the co-delivery of vimentin siRNA in combination with chemotherapy in vivo.

To our knowledge, our present studies is the first one demonstrating that inhibition of vimentin protein expression by vimentin-specific siRNA could be increasing an induction of apoptosis in tumors together with DOX, and also significantly suppress tumors growth in vivo. In results of co-delivery of DOX and vimentin siRNA by galactosylated liposome, we have showed that it is feasible to use a combined-delivery system to deliver nucleic acid and drug both in vitro and in vivo.

Ligand-mediated targeting for drug delivery systems is one of the most exciting areas because it can improve site-specific drug delivery. Many attempts have been made to deliver drugs specifically to liver cells for effective treatment of liver diseases. Among the various receptors on parenchymal and nonparenchymal cells, the ASGPR is the most promising receptor site because of its high affinity and rapid internalization [26]. Targeting ASGPR is an attractive strategy for liver-specific delivery due to its exclusive expression by parenchymal hepatocytes [27]. In order to achieve highly effective hepatic tumor-targeting delivery systems for gene and chemotherapeutic agents, Gal-liposome containing a galactosylated lipid (Gal-ceramide) and a cationic lipid (DMKE) was designed and evaluated for targeting and delivery efficiency.

In this study, the hypothesis that the ASGPR targeted Gal-siRNA-L shows better suppression efficacy than free siRNA can be evidenced by the anti-cancer effects and cell uptake effects between these experimental conditions were similar in vitro (Figure 4). The cytotoxicity of different DOX formations was also studied by MTT assay, which measures mitochondrial function or integrity. DOX proved to be an excellent compound for demonstration of targeted drug delivery. The fluorescence properties of DOX allow for quantitative measurement of biodistribution both by determining the DOX concentration in tissues by its fluorescence intensity at 495 nm measured using fluorescence spectrophotometer (Figure 6a). The biodistribution study results suggest that Gal-DOX/siRNA-L allows higher DOX concentrations to be reached in both normal liver and hepatic tumor tissue than do DOX/siRNA-L (non-targeted liposomes) and free DOX. We also observed in our study that both the Gal-DOX/siRNA-L and DOX/siRNA-L liposomes concentrations were much higher in livers. This result is consistent with previously published studies showing that the cationic liposomes are preferentially uptaken by the liver. The first possible reason is that the macrophages or Kupffer cells in the liver facilitated the uptake, and consequently stayed the liposomes in hepatocytes [28]. The second possible cause is that the extensive blood vessel (including capillary) in the liver facilitated the uptake of liposomes by the hepatocytes. This phenomenon may enhance that this delivery system is possibly appropriate for the treatment of hepatocarcinoma. On the other hand, in the heart, Gal-DOX/siRNA-L showed a lower drug concentration than DOX/siRNA-L and free DOX. This result suggests that the galactosylated liposome has selective hepatic tumor targeting.

For targeted nano-liposomes, a linked targeting ligand can significantly improve the therapeutic efficacy of chemotherapy or molecular therapy in cancer. Our data showed that the Gal-DOX/siRNA-L could efficiently bind to Huh7 cells overexpressing ASGPR and increase the uptake of DOX and vimentin siRNA, suggesting the effectiveness of ASGPR targeting. On the other hand, the targeting activity of a galactose-conjugated liposome is highly dependent on the asialoglycoprotein receptors (ASGPR), which may limit the application of ASGPR targeting liposome delivery. Importantly, our data demonstrated the rational of co-delivery of DOX and vimentin siRNA since the selection of chemotherapeutics or gene drugs is important for combined therapy. Both in vitro and in vivo results illustrated that these two drugs enhanced effects. Consistent with its higher accumulation in tumor tissue, Gal-DOX/siRNA-L had stronger tumor growth inhibition activity than DOX/siRNA-L and free DOX (Figure 6b), which may be explained by the following: galactosylation of the liposomes enhanced cellular uptake and internalization of DOX/siRNA into hepatoma cells via ASGPR-mediated pathways; PEGylation of liposomes increased accumulation of DOX in tumor tissue by prolonging the circulation time and reducing uptake by the RES; the small particle size of Gal-DOX/siRNA-L enables easier penetration of the tumor via the enhanced permeation and retention effect.

Insufficient therapeutic efficacy due to low cancer target approaching, and the induction of side effects by non-selectivity into normal cells and the over-dose use of chemotherapeutic agents by drug-resistance still act as a variety of obstacles for successful cancer therapy. In order to overcome these disadvantages, it is to use the combined therapeutic method capable of inducing cancer cell killing by different mechanisms.

At this point, the synergistic or combined effects by co-delivering anti-cancer drugs and siRNAs have been verified by other studies using different delivery system or targeting genes. Chen et al. designed a CD13 targeting (NGR peptide) PEGylated LPD (liposome-polycation-DNA) nanoparticle (LPD-PEG-NGR) to deliver both doxorubicin and c-MYC siRNA, and they reported the enhanced cancer therapeutic effects in tumor mouse model [29]. Some research groups also applied novel material to deliver chemotherapeutic drugs and siRNA, such as nanostructured lipid carriers (NLCs) [30,31]. When these delivery systems were used for co-delivery anti-cancer drugs and siRNA, enhanced anti-cancer effects were achieved as the liposome based drug delivery system dose. Even though our combination system was not examined in drug-resistant cancer cells to demonstrate the superiority of more exact therapeutic effects, we delineate that the combination strategy of gene therapy and chemotherapy elicits increased anti-cancer effects. These studies, together with our study, collectively suggest that the combined effect by co-delivering chemotherapeutic drugs and siRNA to cancer cells is a universal phenomenon and holds promise for future cancer treatments. Our next goal will be focused on the therapeutic effects by combination treatment in drug-resistant cancer cells.

In summary, a novel galactosylated liposomal vehicle modified with galactosylated-ceramide and DMKE lipids was developed for co-delivering chemotherapeutic drugs and siRNA to hepatoma cells. Therefore, this novel galactosylated liposomal formulation is prospective as a targeted carrier for anti-cancer drugs against hepatic tumor and preliminary investigation.

## 4. Materials and Methods

### 4.1. Materials

d-Galactosyl ceramide, PEG-DSPE (1,2-distearoyl-*sn*-glycero-3-phosphoethanolamine (ammonium salt)), cholesterol, POPC (1-palmitoyl-2-oleoyl-*sn*-glycero-3-phosphocholine), rhodamine-conjugated DOPE (1,2-dioleoyl-sn-glycero-3-phosphoethanolamine), and DOTAP (dioleoyl-3-trimethylammonium-propane) were purchase from Avanti Polar Lipid, Inc. (Alabaster, AL, USA). The cationic lipid DMKE (*O*,*O*’-dimyristyl-*N*-lysyl glutamate) was synthesized as previously reported [32]. EX-CyTox that is cell viability and cytotoxicity test reagent is Daeil Lab Service Co., Ltd (Seoul, South Korea). Doxorubicin and CL-4B (agarose beads) were purchased from Sigma-Aldrich Chemical (St. Louis, MO, USA). FITC-labeled siRNA was purchased from Invitrogen^™^ Life Technologies (Grand Island, NY, USA). siRNA against vimentin was obtained from Santa Cruz biotechnology (Dallas, TX, USA).

### 4.2. Preparation of Galatosylated Liposomes

Cationic liposomes were prepared as described previously with a slight modification [33]. Briefly, to prepare the galactosylated cationic liposome, DMKE, cholesterol, β-d-galactosyl ceramide, POPC, and PEG_2000_-DSPE were mixed in a molar ratio of 2:1:1:1:0.2 in chloroform:methanol (2:1, v/v). The chloroform and methanol were evaporated under a stream of N_2_ gas, and vacuum desiccated for a minimum of 1 h to ensure removal of residual organic solvent. The dried lipid films (1 mg total lipids) were hydrated with 1 mL of citrate buffer (20 mM citiric acid (pH 4.0) and 150 mM NaCl) with a vortex mixer for 5 min. The hydrated lipids (1.0 mg/mL) were sonicated with a bath-type sonicator (Branson Inc. Danburry, CT, USA) three times for 30 s with a 10-s interval, which produced small unilamellar liposomal vesicles. The liposome solution was then extruded through 800-, 400-, 200-, and 100-nm pore size polycarbonate membrane (Merck Millipore, Billerica, MA, USA) to serially decrease the extrusion size.

### 4.3. Preparation of DOX-Encapsulating Galactosylated Liposomes (Gal-DOX-L) and Determination of DOX Loading Efficiecy

To encapsulate DOX into the liposomes, the pH gradient-driven remote loading method was employed. Briefly, galactosylated liposomes were prepared using the same method described above. The buffer solution containing the liposomes was then exchanged with HEPES (4-(2-hydroxyethyl)-1-piperazineethanesulfonic acid) buffer (pH 7.4) by CL4B column chromatography. To introduce DOX into the liposomes, one milliliter of the liposomal solution (1 mg/mL) was incubated with 150 μL of DOX (2 mg/mL) for 10 min at 60 °C via the difference in pH between the inside and the outside of the liposomes. The concentration of DOX in the Gal-DOX-L calculated by measuring the absorbance at 492 nm and using a standardization curve generated for DOX quantification. For determination of DOX loading efficiency in galactosylated liposome, quantification of DOX within Gal-DOX-L was determined by measuring fluorescence excitation and emission using a plate reader (iMark^™^ Microplate Absorbance Reader, Bio-Rad) set at 480 and 580 nm after incubation in 1% Triton X-100 solution for 45 min.

### 4.4. Preparation of Gal-DOX/siRNA-L Complexes

Gal-DOX/siRNA-L complexes were prepared by the charge interaction with different nitrogen to phosphate (N/P) ratios (1, 3, 6, and 9). Briefly, the Gal-DOX/siRNA-L complexes were prepared at an N/P (liposome/siRNA) ratio of 9:1 in RNase free H_2_O by adding stock solution of Gal-DOX-L into a prepared 5 nmol of siRNA solution. After the samples were gently vortexed, and the solutions were incubated for 30 min at room temperature to facilitate efficient incorporation of siRNA. The loading efficiency of siRNA by Gal-DOX-L was determined by the fluorescence of FITC-labeled siRNA (or ethidium bromide (EB) dye) displacement assay in the agarose gel. Unconjugated siRNA will migrate in the gels, but the complexed siRNA will be stay in the loading wells with liposomes because of the neutral charge of lipoplexes.

### 4.5. Physicochemical Characterization

The size and zeta-potentials of the liposomes (Gal-DOX-L) and lipoplexes (Gal-DOX/siRNA-L) were measured using a Zetasizer (Nano ZS, Malven, UK). The lipoplexes were prepared by mixing different N/P ratios of cationic liposomes and siRNA. All of the measurements were carried out at 25 °C. Each parameter was checked three times and means and standard deviations were calculated.

Agarose gel retardation studies were used to evaluate the siRNA loading in liposomes. The samples containing 200 nM of FITC-siRNA, with varying N/P ratios in nuclease free water, were applied to a 2% (w/v) agarose gel in TAE buffer. The free siRNA or unconjugated siRNA will migrate in the gels but the complexed siRNA will be stuck in the loading wells with liposomes. The encapsulation efficiency (EE) of siRNA in Gal-DOX/siRNA-L complexes were determined the integrity of the preserved Gal-DOX/siRNA-L compared to free siRNA. The fluorescence intensities measured uncomplexed free FITC-labeled siRNA at varying N/P ratios in the gel by FUSION-SL chemiluminescence analyzer and software (VILBER, Suarlée, Belgium).

### 4.6. Cell Culture and Assay for Cell Binding of Gal-DOX-L

Huh7 cells, a human hepatoma cell line, and A549 cells, a human lung cancer epithelial cell line, were cultured in Dulbecco’s Modified Eagle’s Medium (DMEM) supplemented with 100 U of penicillin, 100 mg of streptomycin, and 10% fetal bovine serum (FBS). To monitor the cell binding of Gal-DOX-L under a fluorescence microscopy, Huh7 cells and A549 cells (1 × 10^5^ cells, each) were seeded into 24-well plates (SPL Life Sciences, Seoul, Korea) in DMEM supplemented with 10% FBS at 37 °C under 5% CO2 for 24 h. After 24 h of incubation, the cells were treated with fresh medium (500 μL) containing DOTAP/cholesterol-liposomes (as a control) or Gal-DOX-Ls (Galactose linked-cationic liposomal doxorubicin) for 15 min in culture media. After the treated cells were washed three times with PBS (phosphate-buffered saline), fresh media was added. These liposomes (containing an amount equivalent to 0.2 μM DOX, each) having the rhodamine-conjugated DOPE lipids (red color) were observed on the cell surface by a fluorescence microscope (JuLi-Smart Fluorescence Cell Imager, NanoEnTek Inc., Seoul, Korea).

### 4.7. In Vitro Cytotoxicity and Cell Uptake Studies

The in vitro cytotoxicity of DOX- or/and vimentin siRNA-loaded liposomes was evaluated by MTT assay method using EZ-CyTox reagents after 48 h. Briefly, A549 cells (5.0 × 10^3^ cells/100 μL per well) and Huh7 cells (5.0 × 10^3^ cells/100 μL per well) were seeded into 96-well plates in DMEM medium supplemented with 10% v/v fetal bovine serum and with 100 IU of penicillin, 100 μg/mL of streptomycin. Cells were incubated with 100 μL of free DOX (0.2 μM), free vimentin siRNA (200 pmole), Gal-DOX-L [DOX, 0.2 μM], Gal-Lipoplexes [vimentin siRNA, 200 pmole], or Gal-DOX/siRNA-L (containing with DOX concentration of 0.2 μM and vimentin siRNA concentration of 200 pmole) for 2 h at 37 °C. As a control liposome, cells were also treated with liposomal doxorubicin (DOX-L [0.2 μM]) lacking of galactose or lipoplexes lacking of galactose (vimentin siRNA, 200 pmole) under the same conditions. After incubation, the cells were washed three times with PBS and cultured for additional 48 h in 200 μL of fresh medium. After 48 h post-transfection, 10 μL of the Ez-cytox reagent (5 mg/mL) was added to each well, followed by incubation for 4 h at 37 °C. The absorbance was measured at 450 nm using a VersaMax ELISA (enzyme-linked immunosorbent assay) microplate reader (Molecular Devices, Sunnyvale, CA, USA).

The cell uptake for siRNA transfection of galatosylated-cationic liposome devoid of DOX was evaluated in vitro on Huh7 cells expressing asialoglycoprotein surface receptors in comparison to free siRNA. Huh7 cells were treated with galactose conjugated cationic lipoplexes with different FITC-labeled siRNA concentration formations of N/P ratio = 9 for 2 h at 37 °C. Next, to determine the transfection efficacy in the effectiveness of siRNA delivery, the treated cells were observed using a general fluorescence microscope. To evaluate the cellular uptake efficiency of FITC-siRNA, the transfected cells were washed with PBS, and treated with Trypsin-EDTA (0.25%) at 37 °C for 5 min. Then, the harvested cells after wash with PBS were observed FITC-siRNA in cells using a confocal laser scanning microscope (FV-1000, Olympus, Tokyo, Japan).

### 4.8. Western Blot Analysis

Western blot analyses for the expression of vimentin were performed using standard methods. In brief, Huh7 cell treated vimentin siRNA pallets were lysed in RIPA (Radioimmunoprecipitation assay) lysis and extraction buffer (Thermo Fisher Scientific, Waltham, MA, USA). The cell lysate was centrifuged at 10,000× *g* for 10 min at 4 °C and then the supernatant was transferred to a fresh 1.5 mL microcentrifuge tube. Protein concentration was determined by the Protein Assay Kit (Pierce Biotechnology, Inc., Rockford, IL, USA) and 20 μg protein samples were loaded per lane of SDS-PAGE gel. These proteins were separated by 10% SDS-PAGE and electrotransferred onto a polyvinylidene difluoride membrane (Merck-Millipore, Billerica, MA, USA). The membrane was blocked with 5% non-fat milk in Tris-buffered saline with Tween 20 (TBST) buffer (20 mM Tris, 150 mM NaCl and 0.1% Tween-20 (pH 7.5)) for 2 h and incubated with the primary antibodies overnight at 4 °C. The dilution of vimentin antibody (mouse monoclonal; Santa Cruz Biotechnology, Inc., Dallas, TX, USA) and β-actin antibody (mouse monoclonal; Santa Cruz Biotechnology, Inc., Dallas, TX, USA) was 1:1000. After the treatment of primary antibodies, the membranes were washed three times with TBST and incubated with horseradish peroxidase (HRP)-conjugated secondary antibody goat anti-mouse IgG (1:5000; Santa Cruz Biotechnology, Inc., Dallas, TX, USA) at room temperature for 1.5 h. The bands were visualized using an ECL Plus Western Reagent (Santa Cruz Biotechnology, Inc., Dallas, TX, USA). The FUSION-SL chemiluminescence analyzer system (VILBER, Suarlée, Belgium) was used to capture images of the blots. The intensity of each band was quantified by densitometry and normalized to that controlled β-actin.

### 4.9. Tissue Distribution and Anti-Tumor Efficacy Study in Vivo

Biodistribution studies were performed on healthy male Balb/c athymic nude mice (eight-weeks of age, 16–18 g) which were purchased from Orient Bio Inc. (Seongnam, Korea). The mice were retained in conformity with the National Institute of Toxicological Research of the Korea Food and Drug Administration guidelines as well as the regulations for the care and use of laboratory animals of the Animal Ethics Committee at Konyang University (No. 0356). All of the animal studies were conducted under protocols approved by the Committee on Use and Care of Animals at Konyang University, Korea. For the tissue distribution assay, Huh7 human hepatoma cells were implanted by subcutaneous inoculation of 3.0 × 10^6^ Huh7 cells into the left flank of male athymic nude mice. At the tumor volume of 400 mm^3^, randomly dividing the tumor-bearing mice into various three groups (four per group) were injected with free drug (DOX), DOX/siRNA-L, or Gal-DOX/siRNA-L at a single dose of 150 μg/kg vimentin siRNA and/or 5 mg/kg DOX via the tail vein. The mice were sacrificed, and then the tumor and major organs (liver, kidneys, spleen, lungs, and heart) were collected at 4 h after drug administration. Then these tissues were washed using a cool PBS and excess fluid was removed. To measure concentration of DOX content in the tissues, each tissue was determined as described in the literature [34]. In briefly, 1.5 mL nuclear lysis buffer (10 mM HEPES, 1 mM MgSO_4_, 1 mM CaCl_2_, pH 7.4) was added to the 0.2–0.3 g piece of tissue samples and then the tissues were homogenized using a Tissue-Tearor homogenizer (BioSpec Products Inc, Bartlesville, OK, USA). The homogenate (100 μL) was treated with 50 μL of 10% (v/v) Triton X-100, 100 mL of DW, and 750 μL acidified isopropanol. The mixture was placed overnight at ‒20 °C, and centrifuged immediately at 14,000 rpm for 10 minutes after thawing. DOX in the supernatant was measured by fluorescence spectrophotometer at fluorescence intensity at 495 nm (Hitachi F-2700, software FL solution, Tokyo, Japan).

For the antitumor activity study, 3.0 × 10^6^ Huh7 cells were injected in the left flank of male athymic nude mice. At the tumor volume of ~55 mm^3^, liposomes or lipoplexes (DOX/siRNA-L, Gal-DOX-L, Gal-Lipoplexes [siRNA], or Gal-DOX/siRNA-L), free drug, or saline was administrated by a single dose tail vein injection (150 μg/kg vimentin siRNA and/or 5 mg/kg DOX) every seven days. The tumor volumes were calipers and calculated using the following formula: (width)^2^ × length/2.

### 4.10. Statistical Analysis

All results are expressed as the mean ± standard deviation (SD) of at least three experiments. Significant differences were determined by *t*-test using SPSS Statistics Premium 21 software (IBM, Armonk, NY, USA). A *p*-value of <0.01 and <0.05 were considered to represent a statistically significant difference.

## 5. Conclusions

In the present study, we have developed and characterized a novel, lipid-based nanocarrier for chemotherapeutic drug and siRNA co-delivery, which can target hepatoma cells. The galactosylated liposomal doxorubicin vehicle (Gal-DOX-L) and gal-doxorubicin/vimentin siRNA liposome (Gal-DOX/siRNA-L) had the correct size (<100 nm and <140 nm, each), delivered DOX and siRNA into hepatoma cells showing dose-dependent efficacy. Furthermore, when Gal-DOX/siRNA-Ls were injected to mice; Gal-DOX/siRNA-L allows higher DOX concentrations to be reached in hepatic tumor tissue than do DOX/siRNA-Ls (non-targeted liposomes) and free DOX. The synergistic or combined effects by co-delivering anti-cancer drugs and siRNAs have also confirmed that Gal-DOX/siRNA-Ls had stronger tumor growth inhibition activity than DOX/siRNA-Ls and free DOX. This novel galactosylated liposome for co-delivery will enable its use as a targeted carrier against hepatic tumor.

## Figures and Tables

**Table 1:** Physicochemical properties of doxorubicin and siRNA in sterically-stabilized galactosylated-liposomes.

**Formulation**	**Particle Size (nm)**	**Zeta Potential (mV) in Water**	**DOX-to-Liposome Ratio (w/w)**	**DOX EE (%)**
Gal-DOX-L	98.4 ± 2.2	35.7 mV	0.3:1	95 ± 1.8
**Formulation**	**Particle Size (nm)**	**Zeta Potential (mV) in Water**	**siRNA Loading (nmol/mL)**	**siRNA EE (%)**
Free siRNA	2.3 ± 1.2	−43.5 mV	5	-
Gal-DOX/siRNA-L (N/P = 1)	125.22 ± 13	3.5 mV	5	21 ± 3.2
Gal-DOX/siRNA-L (N/P = 3)	127.54 ± 11	6.4 mV	5	56 ± 2.5
Gal-DOX/siRNA-L (N/P = 6)	137.45 ± 13	7.3 mV	5	65 ± 3.2
Gal-DOX/siRNA-L (N/P = 9)	135.21 ± 22	9.4 mV	5	86 ± 2.3

Values are mean ± SD. EE: encapsulation efficiency.

## Supplementary Materials

The following are available online at http://www.mdpi.com/2079-4991/6/8/141/s1.

## Abbreviations

ASGPR	asialoglycoprotein receptors
Chol	cholesterol
DMEM	Dulbecco’s modified Eagle medium
DMKE	*O,O*′-dimyristyl-*N*-lysyl glutamate
DOPE	1,2-dioleoyl-*sn*-glycero-3-phosphoethanolamine
DOTAP	dioleoyl-3-trimethylammonium-propane
DOX	doxorubicin
DSPE	distearoyl phosphatidyl ethanolamine
EE	encapsulation efficiency
FBS	fetal bovine serum
MDR	multi-drug resistance
PEG	polyethylene glycol
POPC	1-palmitoyl-2-oleoyl-*sn*-glycero-3-phosphocholine
PBS	phosphate buffered saline
siRNA	small interfering RNAs

## References

[1] Li J., Wang Y., Zhu Y., Oupický D. (2013). Recent advances in delivery of drug-nucleic acid combinations for cancer treatment. J. Control. Release.

[2] Biju V. (2014). Chemical modifications and bioconjugate reactions of nanomaterials for sensing, imaging, drug delivery and therapy. Chem. Soc. Rev..

[3] Fortier C., Durocher Y., De Crescenzo G. (2014). Surface modification of nonviral nanocarriers for enhanced gene delivery. Nanomedicine.

[4] Kirtane A.R., Panyam J. (2013). Polymer nanoparticles: Weighing up gene delivery. Nat. Nanotechnol..

[5] Gewirtz D.A. (1999). A critical evaluation of the mechanisms of action proposed for the antitumor effects of the anthracycline antibiotics adriamycin and daunorubicin. Biochem. Pharmacol..

[6] Ameyar-Zazoua M., Guasconi V., Ait-Si-Ali S. (2005). siRNA as a route to new cancer therapies. Exper. Opin. Biol. Ther..

[7] Kim J.S., Lee Y.K., Jeong H.Y., Kang S.J., Kim M.W., Ryu S.H., Kim H.S., Kim K.S., Kim D.E., Park Y.S. (2013). Sendai F/HN Viroplexes for efficient transfection of leukemic T cells. Yonsei Med. J..

[8] Chen A.M., Zhang M., Wei D., Stueber D., Taratula O., Minko T., He H. (2009). Co-delivery of doxorubicin and Bcl-2 siRNA by mesoporous silica nanoparticles enhances the efficacy of chemotherapy in multidrug-resistant cancer cells. Small.

[9] Saad M., Garbuzenko O.B., Minko T. (2008). Co-delivery of siRNA and an anticancer drug for treatment of multidrug-resistant cancer. Nanomedicine (Lond.).

[10] Rahman A., Husain SR., Siddiqui J., Verma M., Agresti M., Center M., Safa A.R., Glazer R.I. (1992). Liposome-mediated modulation of multidrug resistance in human HL-60 leukemia cells. J. Natl. Cancer Inst..

[11] Gabizon A., Shmeeda H., Horowitz A.T., Zalipsky S. (2004). Tumor cell targeting of liposome-entrapped drugs with phospholipid-anchored folic acid-PEG conjugates. Adv. Drug Deliv. Rev..

[12] Perez A.T., Domenech G.H., Frankel C., Vogel C.L. (2002). Pegylated liposomal doxorubicin (Doxil) for metastatic breast cancer: The Cancer Research Network, Inc., experience. Cancer Investig..

[13] Theodoulou M., Hudis C. (2004). Cardiac profiles of liposomal anthracyclines: Greater cardiac safety versus conventional doxorubicin?. Cancer.

[14] Wu J., Lu Y., Lee A., Pan X., Yang X., Zhao X., Lee R.J. (2007). Reversal of multidrug resistance by transferrin-conjugated liposomes co-encapsulating doxorubicin and verapamil. J. Pharm. Pharm. Sci..

[15] Xiang G., Wu J., Lu Y., Liu Z., Lee R.J. (2008). Synthesis and evaluation of a novel ligand for folate-mediated targeting liposomes. Int. J. Pharm..

[16] Xiong X.B., Huang Y., Lu W.L., Zhang X., Zhang H., Nagai T., Zhang Q. (2005). Enhanced intracellular delivery and improved antitumor efficacy of doxorubicin by sterically stabilized liposomes modified with a synthetic RGD mimetic. J. Control. Release.

[17] Kim K.S., Lei Y., Stolz D.B., Liu D. (2007). Bifunctional compounds for targeted hepatic gene delivery. Gene Ther..

[18] Thapa B., Kumar P., Zeng H., Narain R. (2015). Asialoglycoprotein receptor-mediated gene delivery to hepatocytes using galactosylated polymers. Biomacromolecules.

[19] Craparo E.F., Sardo C., Serio R., Zizzo M.G., Bondì M.L., Giammona G., Cavallaro G. (2014). Galactosylated polymeric carriers for liver targeting of sorafenib. Int. J. Pharm..

[20] Zhou X., Zhang M., Yung B., Li H., Zhou C., Lee L.J., Lee R.J. (2012). Lactosylated liposomes for targeted delivery of doxorubicin to hepatocellular carcinoma. Int. J. Nanomed..

[21] D’Souza A.A., Devarajan P.V. (2015). Asialoglycoprotein receptor mediated hepatocyte targeting—Strategies and applications. J. Control. Release.

[22] Li Y., Huang G., Diakur J., Wiebe L.I. (2008). Targeted delivery of macromolecular drugs: Asialoglycoprotein receptor (ASGPR) expression by selected hepatoma cell lines used in antiviral drug development. Curr. Drug Deliv..

[23] Thiery J.P. (2002). Epithelial-mesenchymal transitions in tumour progression. Nat. Rev. Cancer.

[24] Langa F., Kress C., Colucci-Guyon E., Khun H., Vandormael-Pournin S., Huerre M., Babinet C. (2000). Teratocarcinomas induced by embryonic stem (ES) cells lacking vimentin: An approach to study the role of vimentin in tumorigenesis. J. Cell Sci..

[25] Havel L.S., Kline E.R., Salgueiro A.M., Marcus A.I. (2015). Vimentin regulates lung cancer cell adhesion through a VAV2–Rac1 pathway to control focal adhesion kinase activity. Oncogene.

[26] Jain N.K., Jain S.K. (2010). Development and in vitro characterization of galactosylated low molecular weight chitosan nanoparticles bearing doxorubicin. AAPS PharmSciTech.

[27] Bernardes G.J., Kikkeri R., Maglinao M., Laurino P., Collot M., Hong S.Y., Lepenies B., Seeberger P.H. (2010). Design, synthesis and biological evaluation of carbohydrate-functionalized cyclodextrins and the liposomes for hepatocyte-specific targeting. Org. Biomol. Chem..

[28] Wang Y., Gao S., Ye W.H., Yoon H.S., Yang Y.Y. (2006). Co-delivery of drugs and DNA from cationic core-shell nanoparticles self-assembled from a biodegradable copolymer. Nat. Mater..

[29] Chen Y., Wu J.J., Huang L. (2010). Nanoparticles targeted with NGR motif deliver c-myc siRNA and doxorubicin for anticancer therapy. Mol. Ther..

[30] Whitehead K.A., Sahay G., Li G.Z., Love K.T., Alabi C.A., Ma M., Zurenko C., Querbes W., Langer R.S., Anderson D.G. (2011). Synergistic silencing: Combinations of lipid-like materials for efficacious siRNA delivery. Mol. Ther..

[31] Taratula O., Kuzmov A., Shah M., Garbuzenko O.B., Minko T. (2013). Nanostructured lipid carriers as multifunctional nanomedicine platform for pulmonary co-delivery of anticancer drugs and siRNA. J. Control. Release.

[32] Kim H.S., Moon J., Kim K.S., Choi M.M., Lee J.E., Heo Y., Cho D.H., Jang D.O., Park Y.S. (2004). Gene-transferring efficiencies of novel diamino cationic lipids with varied hydrocarbon chains. Bioconjug. Chem..

[33] Kim H., Lee K.H., Kim K.B., Park Y.S., Kim K.S., Kim D.E. (2013). Antiviral efficacy of a short PNA targeting microRNA-122 using galactosylated cationic liposome as a carrier for the delivery of the PNA-DNA hybrid to hepatocytes. Korean Chem. Soc..

[34] Laginha K.M., Verwoert S., Charrois G.J., Allen T.M. (2005). Determination of doxorubicin levels in whole tumor and tumor nuclei in murine breast cancer tumors. Clin. Cancer Res..

