# Supplementary material for: Galactosylated Liposomes for Targeted Co-Delivery of Doxorubicin/Vimentin siRNA to Hepatocellular Carcinoma
Source: Nanomaterials (Basel). 2016 Jul 30;6(8):141. doi: 10.3390/nano6080141 (PMC5224624; doi:10.3390/nano6080141)
Supplement: Supplementary file 1 [file nanomaterials-06-00141-s001.pdf]

Supplementary Materials: Galactosylated Liposomes for Targeted Co-delivery of Doxorubicin/Vimentin siRNA to Hepatocellular Carcinoma

Hea Ry Oh, Hyun-Young Jo, James S. Park, Dong-Eun Kim, Je-Yoel Cho, Pyung-Hwan Kim and Keun-Sik Kim

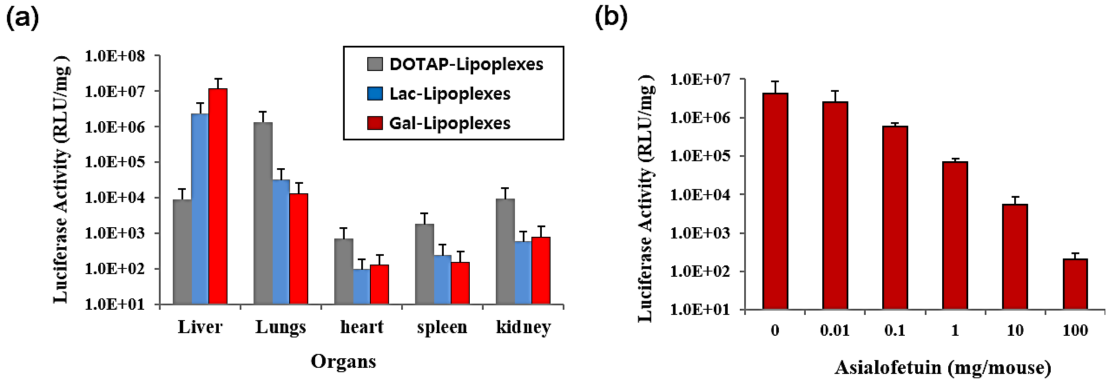

Figure S1. Liver specific gene transfer by Gal-Lipoplexes and dose dependent inhibition by asialofetuin. (**a**) To evaluate the specific transfection to liver in vivo, CD-1 mice (20 g) were injected intravenously with Gal-Lipoplexes (Galactose conjugated liposomal pDNA complexes) or Lac-Lipoplexes (Lactose conjugated liposomal pDNA complexes) solution of 200 µL containing 50 µg of pAAV-CMV-Luc (plasmid AAV vector for luciferase reporter gene expression by a CMV promoter) per mouse ($n = 4$). (**b**). For asialofetuin inhibition, the protein in 100 µL of saline was injected into mice via the tail vein 30 s before the injection of Gal-Lipoplexes (200 µL). Animals were sacrificed 12 h post injection of DOTAP Lipoplexes, Gal-Lipoplexes, or Lac-Lipoplexes, and then the liver, lungs, heart, spleen and kidneys removed. Individual organs were placed into lysis buffer (0.1 M Tris, 0.1% Triton X-100, 2 mM EDTA (ethylenediaminetetraacetic acid), pH 7.8) in a ratio of 4–8 mL buffer per mg of the wet tissue. Each tissue sample was homogenized for 20–30 s with a Tissue Tearor and centrifuged for 10 min at 12,000 rpm at 4 °C. Protein concentration and luciferase activity in tissue extracts were determined.

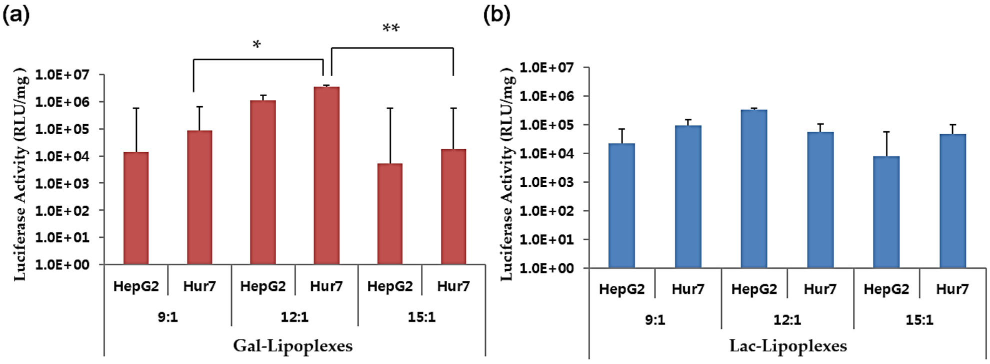

Figure S2. Optimization of weight ratio for transfection efficiency of hepatocyte targeted Lipoplexes by galactose or lactose ligands in hepatoma cells. Gal-Lipoplexes or Lac-Lipoplexes were added to Huh7 or HepG2 cells. Also 1 µg of pDNA (pAAV-CMV-Luc) complexed to cationic liposome (Galactosylated liposome (a) or Lactosylated liposome (b)) was added to each hepatoma cells. The cells were transfected for 4 h and incubated for an additional 24 h. The luciferase activity of the cell lysate was measured with a luminometer by luciferase assay method. Each bar represents mean ± SEM for four independent experiments. * $p < 0.05$, ** $p < 0.01$ when compared with 9:1 Hur7 group or 15:1 Hur7 group.

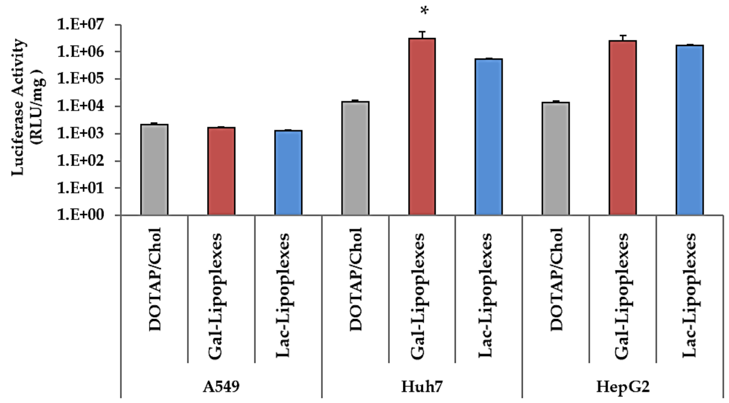

Figure S3. Transfection efficiency of hepatocyte targeted liposomes including galactose or lactose ligands. [1]DOTAP/Chol and hepatocyte targeted liposomes (galactose conjugated liposome or lactose conjugated liposomal) to were added to A549 (control cell), Huh7, and HepG2 cells. Also 1 μg of pDNA complexed to each liposomes (1:12 weight ratio of DNA and liposome) was added to these cells. The cells were transfected for 4 h and incubated for an additional 24 h. The luciferase activity of the cell lysate was measured with a luminometer and a luciferase assay kit. The mean luciferase activity was calculated from three different measurements. Each bar represents mean ± SEM (standard error of the mean) for four independent experiments. * $p < 0.05$ when compared with Lac-DPCP group. [1]DOTAP: dioleoyl-3-trimethylammonium-propane

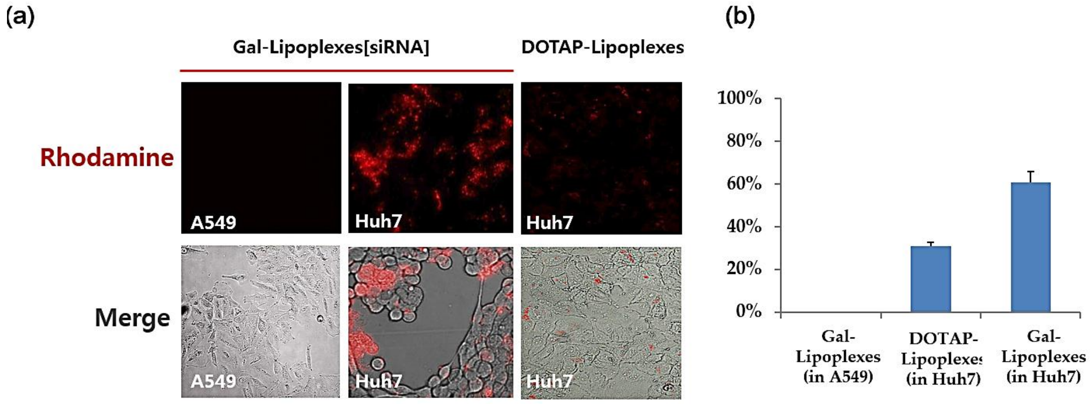

Figure S4. Cellular specific binding of Gal-Lipoplexes[siRNA] to galactose-binding receptor *in vitro*. Galactose-binding receptor (+) cell line (Huh7 cells) was compared with negative controls cell line (A549 cells) after treatment with Gal-Lipoplexes[siRNA] for 20 min, by a fluorescence microscope (JuLI, NanoEnTec, Seoul, South Korea). (**a**) Rhodamine modified Gal-Lipoplexes or DOTAP-Lipoplexes (red color) were only shown the cell specific binding at Huh7 cells. (**b**) Red fluorescence color in each cells were counted by a Tali image-based cytometer (Thermo Fisher Scientific Inc., Waltham, MA, USA).

© 2016 by the authors; licensee MDPI, Basel, Switzerland. This article is an open access article distributed under the terms and conditions of the Creative Commons Attribution) license (http://creativecommons.org/licenses/by/4.0/).
